# Analysis of the interaction between host factor Sam68 and viral elements during foot-and-mouth disease virus infections

**DOI:** 10.1186/s12985-015-0452-8

**Published:** 2015-12-23

**Authors:** Devendra K. Rai, Paul Lawrence, Anna Kloc, Elizabeth Schafer, Elizabeth Rieder

**Affiliations:** Foreign Animal Disease Research Unit, United States Department of Agriculture, Agricultural Research Service, Plum Island Animal Disease Center, USDA/ARS/NAA, P.O. Box 848, Greenport, NY 11944 USA

**Keywords:** Sam68, FMDV, 3C protease, 3D polymerase, IRES, Stress granules, RNA replication

## Abstract

**Background:**

The nuclear protein *S*rc-*a*ssociated protein of 68 kDa in *m*itosis (Sam68) is known to bind RNA and be involved in cellular processes triggered in response to environmental stresses, including virus infection. Interestingly, Sam68 is a multi-functional protein implicated in the life cycle of retroviruses and picornaviruses and is also considered a marker of virus-induced stress granules (SGs). Recently, we demonstrated the partial redistribution of Sam68 to the cytoplasm in FMDV infected cells, its interaction with viral protease 3C^pro^, and found a significant reduction in viral titers as consequence of Sam68-specific siRNA knockdowns. Despite of that, details of how it benefits FMDV remains to be elucidated.

**Methods:**

Sam68 cytoplasmic localization was examined by immunofluorescent microscopy, counterstaining with antibodies against Sam68, a viral capsid protein and markers of SGs. The relevance of RAAA motifs in the IRES was investigated using electromobility shift assays with Sam68 protein and parental and mutant FMDV RNAs. In addition, full genome WT and mutant or G-luc replicon RNAs were tested following transfection in mammalian cells. The impact of Sam68 depletion to virus protein and RNA synthesis was investigated in a cell-free system. Lastly, through co-immunoprecipitation, structural modeling, and subcellular fractionation, viral protein interactions with Sam68 were explored.

**Results:**

FMDV-induced cytoplasmic redistribution of Sam68 resulted in it temporarily co-localizing with SG marker: TIA-1. Mutations that disrupted FMDV IRES RAAA motifs, with putative affinity to Sam68 in domain 3 and 4 cause a reduction on the formation of ribonucleoprotein complexes with this protein and resulted in non-viable progeny viruses and replication-impaired replicons. Furthermore, depletion of Sam68 in cell-free extracts greatly diminished FMDV RNA replication, which was restored by addition of recombinant Sam68. The results here demonstrated that Sam68 specifically co-precipitates with both FMDV 3D^pol^ and 3C^pro^ consistent with early observations of FMDV 3C^pro^-induced cleavage of Sam68.

**Conclusion:**

We have found that Sam68 is a specific binding partner for FMDV non-structural proteins 3C^pro^ and 3D^pol^ and showed that mutations at RAAA motifs in IRES domains 3 and 4 cause a decrease in Sam68 affinity to these RNA elements and rendered the mutant RNA non-viable. Interestingly, in FMDV infected cells re-localized Sam68 was transiently detected along with SG markers in the cytoplasm. These results support the importance of Sam68 as a host factor co-opted by FMDV during infection and demonstrate that Sam68 interact with both, FMDV RNA motifs in the IRES and viral non-structural proteins 3C^pro^ and 3D^pol^.

**Electronic supplementary material:**

The online version of this article (doi:10.1186/s12985-015-0452-8) contains supplementary material, which is available to authorized users.

## Background

Foot-and-mouth disease virus (FMDV) is one of the most contagious animal viruses and causes disease in important livestock species including cattle, pigs, and sheep. The virus is represented by seven different serotypes: A, O, C, Asia1, SAT1, SAT2, and SAT3; each with a distinctive global distribution. Control of FMD outbreaks are typically combated through a combined strategy of mass vaccination and animal culling. Given the profound economic fallout following the spread of FMDV, it is of critical importance to understand the factors that influence FMDV virulence in order to design improved anti-viral strategies [1, 2].

FMDV is the prototypic member of the Aphthovirus genus of the *Picornaviridae* family. It possesses a single-stranded positive-sense RNA genome encoding a single polyprotein that is subsequently cleaved to produced four structural proteins (VP1, VP2, VP3, and VP4) and ten non-structural proteins (L^pro^, 2A, 2B, 2C, 3A, 3B_1,2,3_, 3C^pro^, and 3D^pol^). The genomic region coding for polyprotein is flanked by 5′ and 3′ non-translated regions (NTRs) [2, 3]. Highly structured internal ribosome entry site (IRES) elements in the 5′NTR, allow the initiation of picornaviral polyprotein synthesis, while cap-mediated initiation is simultaneously repressed. IRES first described in picornaviruses [4–6], function via the recruitment of a number of canonical and non-canonical factors [6–10]. IRES are grouped into four types on the basis of their organization and interaction with translation factors and ribosomes. Many host proteins known as IRES *trans*-acting factors (ITAFs) also impact IRES activity. FMDV contains a type II IRES that is predicted to fold into five domains, followed by a polypyrimidine tract and by two alternative AUG initiation codons [11]. GNRA motif in domain 3 is critical to FMDV IRES activity, and mutations in its apical CAAA motif were found to influence IRES activity; however, the function of these two domains in relation to RNA-protein interaction activities awaits further elucidation [12].

In a previous study, we presented evidence suggesting that the 68 kDa Src-associated protein in mitosis (Sam68) host protein binds to the IRES elements of FMDV, which could impact IRES translation activity. We had also shown appearance of Sam68 cleavage products in FMDV-infected cells and in the presence of semi-purified recombinant 3C proteinase (3C^pro^). Interestingly, Sam68 knockdown resulted in a more significant reduction of FMDV titers than its impact in IRES-driven protein translation when using a bicistronic construct [13]. Apart from its role in FMDV infection, multiple reports have described Sam68 as an RNA-binding protein with a role in the replication of other picornaviruses [14–16] as well as retroviruses including the human immunodeficiency virus (HIV) [17–32]. Moreover, Sam68 has been found to be a multi-functional protein affecting a vast array of cellular functions, including RNA-splicing, tumorigenesis, cell cycle progression, apoptosis, adipogenesis, and signal transduction [33–47]. The diversity of functional roles of Sam68 appears to be partly regulated through various post-translational modifications including: tyrosine phosphorylation, serine/threonine phosphorylation, lysine acetylation, arginine methylation, and lysine sumoylation [37, 43, 48–53].

Here, we examined the partial re-localization of Sam68 from the nucleus to the cytoplasm in the context of two relevant host cell lines and provided evidence for Sam68 temporary co-localization with one constituent of stress granules (SGs): T-cell intracellular antigen 1 (TIA-1), in agreement with reports for Sam68 being a stress granule [54] marker for virus-induced cellular stress [16]. Moreover, we investigated the binding of Sam68 to the FMDV IRES, and assessed if UAAA sequence motifs are involved in complex formation. The significance of these interactions to the virus RNA replication was investigated through the depletion and restoration of Sam68 in a cell-free extract system. Consistent with its cleavage and contribution to viral RNA replication, Sam68 was found to co-localize and co-precipitate with FMDV 3C^pro^ and 3D^pol^, highlighting the importance of this host factor to the progression of FMDV infection.

## Results

### Cytoplasmic Sam68 co-localizes with SG markers during FMDV infection

**Fig. 1:**
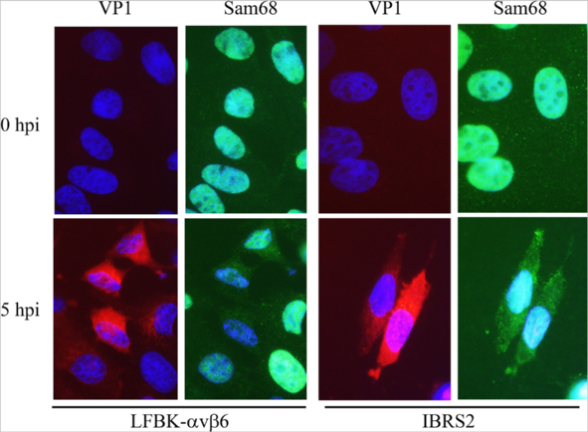
Sam68 redistribution from the nucleus to the cytoplasm. Two different FMDV-susceptible cell lines (LFBK-αvβ6, *left*; and IBRS2, *right*) were mock-infected or infected with FMDV at a MOI of 10 and fixed at 5 hpi. Cells were examined by IFM probing with rabbit polyclonal anti-Sam68 followed by goat-anti-rabbit-AF488 (green) and mouse monoclonal anti-FMDV VP1 followed by goat-anti-mouse-AF568 (*red*). Nuclei were stained with DAPI (*blue*)

In our previous study, FMDV infection was shown to induce a partial redistribution of the nuclear protein Sam68 to the cell cytoplasm in the LFBK cell line [13, 55, 56]. To confirm that this observation could be recapitulated in more widely distributed cell lines, immunofluorescent microscopy was performed using the recently described LFBK-αvβ6 and IBRS2 cell lines. As shown in Fig. 1, Sam68 is exclusively nuclear in mock-infected LFBK-αvβ6 [55] and IBRS2 [57] cell lines. However, in the FMDV-infected cells (visualized by positive reactivity against the FMDV structural protein VP1) Sam68 exhibited partial redistribution to the cytoplasm, and this effect appeared more pronounced with the porcine IBRS2 cells.

**Fig. 2:**
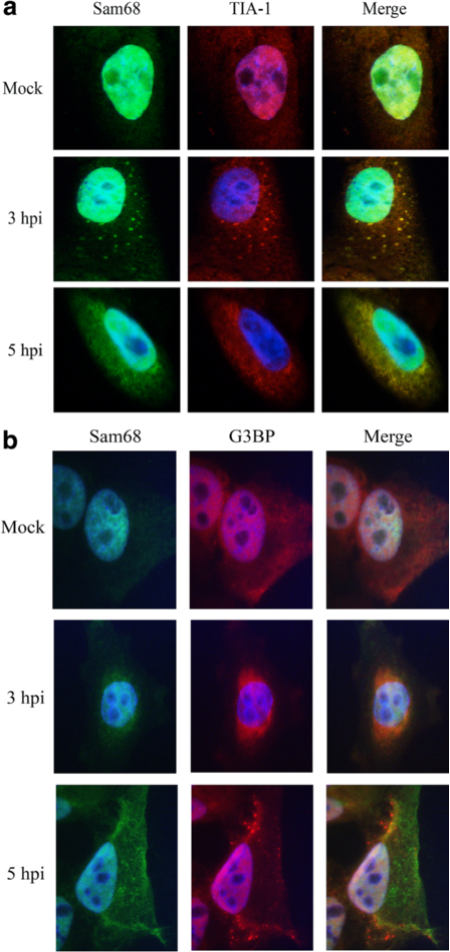
FMDV-induced cytoplasmic Sam68 co-localizes with TIA-1. LFBK cells were mock-infected or infected with FMDV at a MOI of 10 and were fixed at 3 and 5 hpi. Cells were examined by IFM probing with rabbit polyclonal anti-Sam68 followed by goat-anti-rabbit-AF488 (green) and goat polyclonal anti-TIA-1 (**a**) or mouse monoclonal anti-G3BP (**b**) followed by donkey-anti-goat-AF568 (*red*, **a**) or goat-anti-mouse-AF568 (*red*; **b**). Nuclei were stained with DAPI (*blue*)

Next we sought to determine if cytoplasmic Sam68 localized with specific SG markers as reported for poliovirus (PV) [16]. To test this possibility mock-infected or FMDV-infected cells were fixed at various time points post-infection and were evaluated by immunofluorescence microscopy (IFM) using antibodies specific to Sam68 as well as SG markers TIA-1 and Ras-GAP SH3 domain binding protein (G3BP) (Fig. 2). Both Sam68 and TIA-1 display diffuse nuclear fluorescence in mock infected (Fig. 2a) and at 1 h post infection (hpi) with FMDV (data not shown). At 3 hpi, both, cytoplasmic Sam68 and TIA-1 produced visible and overlapping fluorescent puncta in the cytoplasm. Interestingly, by 5 hpi, the specific fluorescent puncta have been replaced with diffuse cytoplasmic fluorescence. As shown in Fig. 2b, overlapping fluorescence can be observed for Sam68 and G3BP at 3 hpi, but unlike TIA-1, the G3BP cytoplasmic fluorescence is diffuse. However, by 5 hpi, punctate cytoplasmic G3BP specific fluorescence is observed, but not in co-localization with Sam68. Interestingly, it has been shown that different Picornaviruses can induce distinct SG compositions, where poliovirus-induced SGs contain Sam68 with G3BP and TIA-1 [16], but cardiovirus-induced SGs see polypyrimidine tract binding protein (PTB) in place of Sam68 [58]. Additionally, herpes simplex virus 2 (HSV-2) was shown to produce cytoplasmic SGs containing G3BP and poly-A binding protein (PABP), and a unique set of nuclear granules containing TIA-1 and Sam68 [59]. Therefore, virus-induced SGs are likely to be compositionally unique. The co-localization of Sam68 with TIA-1 in transient cytoplasmic granular structures in FMDV-infected cells resembles earlier observations in poliovirus and retroviruses [16, 17, 32, 60, 61].

### Examination of Sam68-FMDV IRES complexes by electrophoretic mobility shift Assay and the requirement of Sam68 KH domain

**Fig. 3:**
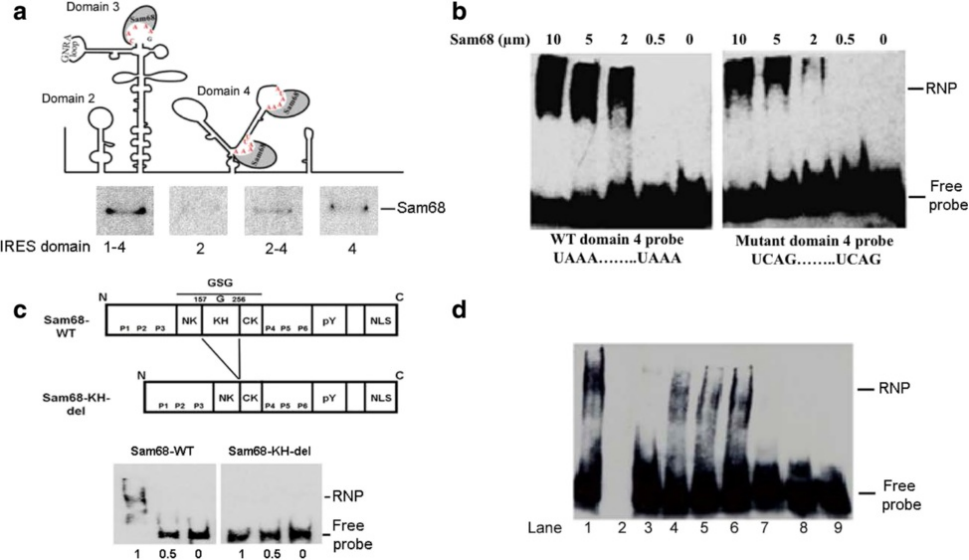
Sam68 interacts with FMDV IRES 4. **a** Cartoon diagram in the upper panel describes the modular structure of FMDV A24 IRES and the location of two unpaired UAAA and a CAAA sequence motifs in domain 4 and 3, respectively. Sam68 potential binding sites are shown as a grey incomplete oval. Lower panel in Fig. 3a shows anti-Sam68 Western blot (rabbit anti-Sam68) of pull-down experiments conducted between Sam68 and selected IRES domains. IRES domains used in the experiment are shown. **b** Determination Sam68 binding to FMDV IRES RNAs by EMSA. WT probe in the left panel consists of 5′ biotin labeled 65 nt long synthetic RNA representing residues 435–499 of FMDV A24-Cru IRES that spanned at least 20 bases upstream and downstream of the two UAAA sequence motifs present in IRES domain 4. The binding of Sam68 to RNA probe was carried out in the presence of 100-fold excess of tRNA. The concentrations of Sam68 used are indicated in each lane. In the mutant probe in the right panel, the two UAAA motifs aremutated to UACG. **c** Upper panel depicts cartoon representation of the wild-type and KH-domain deleted Sam68 constructs. Lower panel shows EMSA results with the addition of Sam68-WT (left) and Sam68-delta KH (right). Probe and conditions used were the same as in section (**b**). **d** Determination of binding interference by various 5′ NTR RNA segments on the complexes formed between WT probe representing partial FMDV IRES domain 4 and Sam68. The binding of Sam68 to WT probe was performed under similar conditions as mentioned in section (**b**) but using a 2 μM Sam68 and 30 nM of probe. WT probe-Sam68 binding was competed with 10-fold molar excess of either full-length IRES (lane 3) or miscellaneous RNAs, including the FMDV cre (lane 4), S-fragment (lane 5), and IRES domains 2, 3, 4 (lanes 6, 7, 8, respectively). Lane 1 contains the binding mixture of Sam68 and domain 4 RNA in the absence of competitor RNAs, whereas lane 9 contains a probe alone control. Lane 2 was left blank

The initiation of cap-independent translation by IRES elements in the 5′ NTR of the picornavirus genome is regulated by RNA-RNA and RNA-protein interactions with canonical and non-canonical translation initiation factors [8–10, 12, 62–66]. Studies on the interaction between Sam68 and FMDV RNA elements indicated that Sam68 binds to IRES region [13]. To define the region within the FMDV IRES involved in this interaction we performed a pulldown experiment using recombinant Sam68 and biotinylated RNAs corresponding to different domains of the FMDV A_24_ IRES (predicted using M-fold software). Figure 3a displays a cartoon diagram of the FMDV IRES [see Background and References] [67, 68] showing the location of RAAA sequences in domain 3 and 4 that could potentially be involved in Sam68 binding. The panel below the cartoon diagram in Fig. 3a displays the pulldown results of Sam68-6H with either full-length IRES (labeled as 1–4) or domain 2, domain 2–4, and domain 4. As evident from the presence of Sam68 reactive bands, domains 4 and 2–4 combined appear to provide Sam68 binding specificity.

To further identify if the two UAAA motifs in domain 4 are involved in binding to Sam68, we prepared 5′-biotin labeled 65 nucleotide (nt) long RNA probes (spanning FMDV A_24_-Cru IRES nt 435–499) encoding the wild-type (WT) sequence or double mutated forms (UAAA to UACG), as described in Material and Methods and Table 1. The M-fold prediction indicated that both the WT and mutant probe maintain the unpaired nature of the two UAAA motifs as observed in the native FMDV IRES. The formation of ribonucleoprotein complex (RNP) between these synthetic RNA probes and Sam68-6H were examined by EMSA in the presence of a 100-fold excess of non-specific competitor tRNA (Fig. 3b). Slower migration of the probe visualized by RNP complex formed in Sam68 and WT domain 4 probe mixtures (Fig. 3b, left panel) was detected in a dose-dependent manner with 50 % binding (Kd_50_) in the range of 2–5 μM, under the experimental conditions. Interestingly, although the mutant domain 4 probe also produced RNP in the presence of Sam68, this binding required approximately 10-fold higher amounts of the protein to produce the same amount of RNP products as with the WT domain 4 probe (Fig. 3b, right panel). Therefore, under the experimental conditions, it appears that the double UACG mutation diminishes Sam68 binding to domain 4 RNA. We believe that the widespread RNP bands observed in EMSA could be due to more than one oligomer form of Sam68 [69].

**Table 1:** Oligonucleotides used in this study

Primer Name	Sequence
P-Q180AForward Sam68	GGGGAAGATTCTTGGACCAGCCGGGAATACAATCAAAAGAC
P-Q180A-Reverse Sam68	GTCTTTTGATTGTATTCCCGGCTGGTCCAAGAATCTTCCCC
P-Q391A-Forward Sam68	GAAGGCTATTACAGCCAGAGTGCCGGGGACTCAGAATATTATGAC
P-Q391A-Reverse Sam68	GTCATAATATTCTGAGTCCCCGGCACTCTGGCTGTAATAGCCTTC
P1579 S IRES -T7	GGATAATACGACTCACTATAGGTTTTCATGAGAAATGGGACGTCTG
P1580 AS IRES	CCTTCCTGTGGCTCGTGGTAGG
P1329 S A_24_-T7cre	GGATAATACGACTCACTATAGGGCTTGAGGAGGACTTG
P1330 AS A_24_-cre	GTCAGTTGGGGAAACCTGCTT
P-21036-IRES loop 1- T7	GATAATACGACTCACTATAGGTTTTCATGAGAAATGGG
P-21037-IRES loop 1 Rev	CTGCTTAGATCGTG
P-1584-IRES loop 2 For-T7	GATAATACGACTCACTATAGGCAAACCGTGCAATTTG
P-1585-IRES loop 2 Rev	GTACAAAGTGTCACCCCTCTAGACCTGG
P-21034-IRES loop 3 For -T7	GGATAATACGACTCACTATAGGGTGTTTGACTCCACGTTCG
P-21035-IRES loop 3 Rev	GCCTGTCACCAGTGCTTGAGTACCAG
P-1581-IRES-4 For -T7	GGATAATACGACTCACTATAGGGACAGGCTAAGGATGCCCTT
P-1582-IRES-4 Rev	ATTCAGACATAGAAGCTTTTTAAACCGGGCG
WT domain 4 probe	5′Biotin-AAGGGGACCGGGGCUUCUAUAAAAGCGCCCGGUUUAAAAAGCUUCUAUGUCUGAAUAGGUGACCG-3′
Mut domain 4 probe	5′Biotin-AAGGGGACCGGGGCUUCUAUACGAGCGCCCGGUUUACGAAGCUUCUAUGUCUGAAUAGGUGACCG-3′
P-2182-Infusion-FMDV A_24_ ires For	GCTTTCCAGGTCTAGAGGGGTGACACTTTGTACTG
P-2183-Infusion-FMDVa_24_ ires Rev	GGTTGTTGGGTCTAGAATAGAACACTTTTCTCTCACC

To further validate the specific binding between Sam68 and IRES, a mutant Sam68 lacking the KH RNA-binding domain (Sam68-KH-del) was expressed and tested side-by-side with full-length protein in an EMSA assay. To this end, EMSA experiments were carried out using WT domain 4 probe (as described above) and either an intact (Sam68-WT) or mutant Sam68 (Sam68-KH-del), (Fig. 3c, upper panel). The EMSA results in the lower panel of Fig. 3c show that the Sam68-WT but not the Sam68-KH-del protein is capable of forming RNP with domain 4 RNA probe.

Having determined a specific interaction between Sam68 and UAAA containing RNA molecule, we next examined if other IRES domains could interfere with the formation of Sam68-domain 4 RNPs. As shown in Fig. 3d, gel shift experiments were carried out using 5′-biotin labeled WT domain 4 probes-Sam68 mixtures either alone (lane 1) or combined with unlabeled transcript RNAs corresponding to: full-length IRES (lane 3), cis-acting replication element (cre, lane 4), the S-fragment (lane 5), IRES domain 2 (lane 6), IRES domain 3 (lane 7), and IRES domain 4 (lane 8) in 10-fold molar excess relative to the probe. Lane 9 contains probe only and lane 2 was left blank. Consistent with earlier observations (Fig. 3a and [13]), the cre (lane 4), S-fragment (lane 5), and IRES domain 2 (lane 6) did not effectively compete with Sam68-domain 4 RNP. For reasons not fully understood, some disruption on RNP signal was detected with the cre, S-fragment, and domain 2 under the current experimental conditions. Nonetheless, IRES domain 3 and 4 that contain the RAAA motif fully disrupted the Sam68-domain 4 RNP complexes, highlighting a major involvement of these IRES elements in RNP formation. As expected, unlabeled full-length IRES (containing domains 1–4) in 10-fold molar excess to the probe also effectively displaced the Sam68-domain 4 RNP complexes.

### Effect of Sam68-depletion on FMDV protein and RNA synthesis using cell-free extracts

**Fig. 4:**
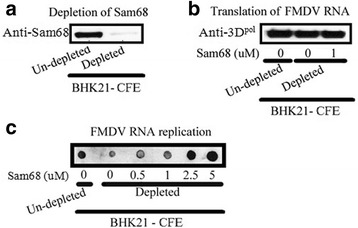
Effect of Sam68-depletion on FMDV protein and RNA synthesis using cell-free extracts. **a** Depletion of Sam68 from BHK-21 CFE. BHK-21 CFE was prepared as described in Materials and Methods. The depletion of Sam68 was confirmed by Western blot probing of non-depleted (*left lane*) and depleted (*right lane*) extract with anti-Sam68. **b** Determination of the effect of Sam68-6H addition on the translation of FMDV A_24_-Cru. FMDV A_24_-Cru RNA was translated using non-depleted or depleted BHK-21 CFE that were supplemented with 0 or 1 μM Sam68-6H as marked. The reaction was carried out at 32 ° C for 2 h and the products were resolved by SDS-PAGE and Western blot probed for FMDV 3D^pol^. **c** Determination of the effect of Sam68-6H addition on the synthesis of FMDV A_24_-Cru RNA. FMDV A_24_-Cru RNA was used for RNA synthesis using non-depleted or depleted BHK-21 CFE that were supplemented with 0–2.5 μM Sam68-6H as marked. The reaction was carried out at 37 ° C for 5 h and the products were SDS-PAGE resolved by dot blotting

In our previous study, we observed that Sam68 knockdown by siRNA led to a significant reduction in virus titers. Intriguingly, the negative impact of this knockdown on virus yields was more pronounced than on the levels of FMDV IRES-driven translation measured with a bicistronic construct [13]. Based on these observations and the results described above, we were compelled to examine the role of Sam68 during FMDV infection in more detail. To this end, we programmed BHK-21 cell-free extracts (CFE) with FMDV RNA to measure protein and RNA synthesis. As shown in Fig. 4, the experiments were conducted using CFE where Sam68 was depleted (using anti-Sam68 antibody) or tested following the addition of Sam68-6H. The Western blot in Fig. 4a shows the depletion of Sam68 in CFE treated with anti-Sam68 antibody directed against the N-terminus of Sam68. In Fig. 4b, 500 ng of transcript FMDV RNA was used to program CFE reactions in Sam68-depleted (0 μM) or supplemented with 1 μM Sam68-6H. Under these conditions, viral protein synthesis (measured by the expression of 3D^pol^) showed no differences between reactions programed with non-depleted, depleted (only) and depleted CFE supplemented with 1 μM Sam68-6H (Fig. 4b). However, we were not able to conclude that the depletion of Sam68 is non-deleterious to viral translation because it could also be argued that other Sam68 splice variants lacking the Sam68 N-terminus were not eliminated in the depleted extracts due to the fact that an N-terminal antibody was used (see Additional file 1: Figure S1). Indeed, there are reports describing Sam68-like molecules, SLM-1 and SLM-2, which have an intact KH-domain but lack the Sam68 N-terminus, may function in binding to the FMDV IRES [70, 71]. A sequence alignment of Sam68 and SLMs shown in Additional file 1: Figure S1 describes the differences between SLMs and full-length Sam68 protein.

Moreover, performing the assay under more stringent conditions, such as lowering the RNA concentrations and/or shortening the incubations times may reveal subtle differences in translation between mutant and parental RNAs. In contrast, in the Sam68-depleted extract a consistent reduction in FMDV RNA synthesis was measured (Fig. 4c). Moreover, addition of Sam68-6H to the depleted CFE displayed a dose-dependent (0.5 to 5 μM) stimulation, evident with increased signal in the dot-blot shown in Fig. 4c. These results provide evidence to suggest a potential involvement of Sam68 host protein in the stimulation of FMDV replication.

### Examination of domain 3 and 4 IRES mutations using FMDV full-length cDNA clones and A_24_ G-luc replicons

**Fig. 5:**
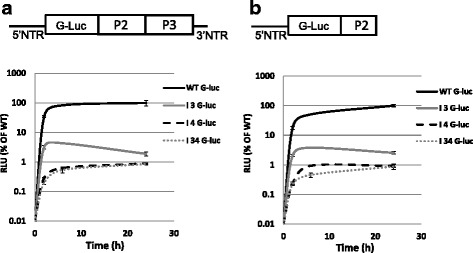
Replication and translation kinetics of WT G-luc, I 3 G-luc, I 4 G-luc and I 34 G-luc replicon constructs. **a** Schematic representation of the G-luc replicon construct digested with SwaI restriction enzyme (*above*). The luciferase activity of transcript RNAs for WT G-luc, I 3 G-luc, I 4 G-luc and I 34 G-luc S*wa*I digested replicon plasmids. On the Y-axis, G-luc signals are expressed in relative luciferase units (RLU) per nanogram of protein and the error bars represent the standard deviation from two independent experiments. X-axis values indicate the time points of G-luc measurement (hours post-transfection). WT, I 3, I 4 and I 34 G-luc are marked as solid black, solid gray, dotted gray and point gray lines, respectively. **b** Schematic representation of truncated G-luc replicon produced by digestion with M*fe*I restriction enzyme (*above*)

To understand the functional significance of Sam68-binding to the FMDV IRES, we next examined the effect of a CAAA to CACG mutation in domain 3 and on two UAAA to UACG mutations in domain 4 using FMDV A_24_Cru infectious cDNA clone and G-luciferase (G-luc) FMDV replicons. The full genome WT or I3 (CAAA to CACG), I4 (two UAAA to UACG), or the two combined (I34) mutant plasmids were first linearized by S*wa*I restriction enzyme digestion and then transcribed into RNA, and electroporated into BHK-21 cells. Twenty-four hours later, the transfected cells were freeze-thawed, and a fraction of the lysate (1/10^th^) was used to initiate next passage. In contrast to the WT virus that exhibited visible cytopathic effect in the second passage in BHK-21 cells, no viable virus was recovered from any of the mutant RNAs even after 7 blind passages (data not shown). This was a surprising observation that led to suspicions about the ability of the mutant RNAs to translate and/or replicate. The same panel of IRES mutations were inserted into FMDV A_24_ G-luc replicons (Fig. 5) to evaluate the importance of these sequence motifs on genome replication and translation. In the G-luc replicon plasmids, the FMDV leader and capsid coding sequences were replaced by gaussia luciferase reporter gene (G-luc, see Methods). Plasmids encoding WT FMDV A_24_ replicon (pA_24_G-luc) or mutant (pA_24_I3 G-luc, pA_24_I4 G-luc and pA_24_I34 G-luc) sequences, were digested with S*wa*I restriction enzyme prior to the RNA transcription. The RNA were then transfected into BHK-21 cells and samples were collected at 0, 2, 6 and 24 h post transfection (hpt) and examined for G-luc expression. As shown in Fig. 5a, the G-luc activity for mutant replicons RNAs was substantially reduced at all time points compared to the WT replicon. At 24 hpt, the G-luc activity for all mutant replicons was less than 5 % of that measured for the WT G-luc. In Fig. 5b, G-luc activity was examined using truncated replicon RNAs obtained by digestion of the pA_24_G-luc plasmids using M*fe*I restriction digests (Fig. 5b, upper panel cartoon). These (G-luc-P2) truncated constructs are lacking 3D^pol^, allowing for measurements of the translation of the input RNA driven by FMDV IRES (WT or mutant). The results in Fig. 5b show that G-luc activity was significantly reduced for mutant (I3, I4, and I34) G-luc replicons compared to WT G-luc RNA, consistent with inhibition of replication observed in Fig. 5a. Together, these results suggest that FMDV IRES function is dependent of the integrity of the RAAA motifs in both domain 3 and 4.

### Sam68 interacts with FMDV 3C^pro^ and 3D^pol^ non-structural proteins

**Fig. 6:**
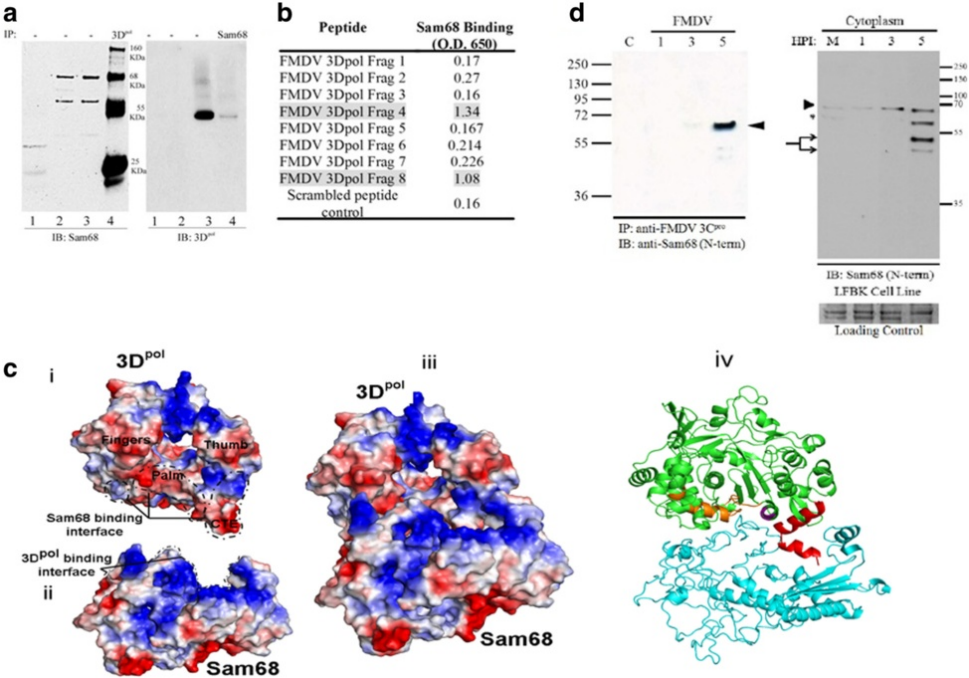
Sam68 interacts with FMDV 3C^pro^ and 3D^pol^. **a** Co-immunoprecipitation of FMDV 3D^pol^ and Sam68 during FMDV infection. BHK-21 cells either mock-infected or infected with FMDV at a MOI of 10 were lysed and the lysates were immunoprecipitated using either anti-FMDV 3D^pol^ or anti-Sam68 and the eluates examined by Western blot. One lane in each panel (as indicated below) was not subject to IP as a control. Equal amount of isotype control antibody served as an IP control. The eluates from the anti-3D^pol^ IP reaction were probed with anti-Sam68 (left panel). Conversely, the Sam68 IP eluates were probed with an anti-3D^pol^ (right panel). In the left panel, lane 1 corresponds to the isotype IP control, lane 2 is mock-infected cell lysate (1:10 dilution), lane 3 is a FMDV-infected cell lysate (1:10 dilution) that was not IP and lane 4 is the anti-3D^pol^ IP eluate from FMDV-infected cell lysates. Similarly, in the right panel, lane 1 corresponds to the isotype control, lane 2 is mock-infected cell lysate (1:10 dilution), lane 3 is a FMDV-infected cell lysate (1:10 dilution) that was not IP, and lane 4 is the anti-Sam68 IP eluate from FMDV-infected cell lysates. **b** The fragments (frag) listed in the table correspond to the amino acid (aa) sequence of FMDV 3D^pol^, starting from the N-terminus: frag #1 aa 1–48, frag #2 aa 49–108, frag #3 aa 109–157, frag #4 aa 158–217, frag #5 aa 218–268, frag #6 aa 269–331, frag #7 aa 332–404, and frag #8 aa 405–470. A scrambled peptide was used as a negative control. **c** Computational prediction of the interaction between FMDV 3D^pol^ and Sam68. (i) Electrostatic surface representation of FMDV 3D^pol^ in the docking pose (PDB: 1U09); red color depicts the negatively charged surface, white shows the neutral surface, and blue color shows the positively charged surface. Color intensity is proportional to the surface charge. Areas under dashed lines indicate Sam68 binding interface of FMDV 3D^pol^. (ii) Electrostatic surface representation of Sam68 in the docking pose. Surface charge and color annotation are same as section (i). Surface marked with dashed lines indicates FMDV 3D^pol^ binding interface of Sam68. (iii) Electrostatic representation of Sam68 docked to FMDV 3D^pol^. [98] FMDV 3D^pol^ green docked on Sam68 blue in cartoon representation. The 3D^pol^ frag-4 residues 193–217 (orange), frag-5 residues 221, 222, 225, 226 (magenta) and frag-8 residues 453–470 (red) form the Sam68 binding interface of 3D^pol^ (**d**) LFBK cells were uninfected or infected with FMDV at a MOI of 10, and cells were harvested at 1, 3, and 5 hpi by treatment with versine. Left panel: cell lysates were IP with mouse monoclonal anti-FMDV 3C^pro^, and examined by Western blot probing with rabbit polyclonal anti-Sam68 (N-terminus). Right panel: collected cells were lysed and separated into nuclear and cytoplasmic fractions, and the cytoplasmic fractions were examined by Western blot probing with rabbit polyclonal anti-Sam68 (N-terminus). Loading control is indicated confirming equivalent loading per lane

Based on the observations related to Sam68 impact on FMDV RNA replication, we carried out experiments to investigate if Sam68 physically interacts with the FMDV RNA-dependent RNA polymerase 3D^pol^ as had previously been shown for poliovirus [14]. To that end, we performed the following experiments: (i) co-immunoprecipitation combined with Western blot analysis in FMDV-infected cells, (ii) indirect enzyme-linked immunosorbent assay (ELISA) performed on Sam68 coated plates using 3D^pol^ peptides binders, and (iii) structural prediction and molecular docking of Sam68 and FMDV 3D^pol^ (Fig. 6).

For the co-immunoprecipitation analysis, BHK-21 cells were either mock infected or infected with FMDV at a MOI of 10 (Fig. 6a). Five hours post-infection, cells were lysed and the lysates were immunoprecipitated [72] with either anti-FMDV 3D^pol^ or anti-Sam68 (N-terminal antibody) crosslinked to protein G Dynabeads. As a control, lysates of virus-infected cells were subject to IP with an isotype antibody control crosslinked to protein G as described above. The left panel of Fig. 6a shows the Western blot of the FMDV 3D^pol^ IP developed with anti-Sam68. In lane 1, a control IP with isotypic antibody shows the absence of a Sam68 specific band. The two faint bands of approximately 20 and 30 kDa are non-specific proteins pulled down by this antibody. Lanes 2 and 3 are the control cell lysates at 1:10 dilution from mock-infected and FMDV-infected cell lysates, respectively. As expected, the endogenous Sam68 as well as the previously described 55 kDa Sam68-like-mammalian protein 2 (SLM-2) [13, 73] were visualized in both control lanes. Lane 4 shows that anti-3D^pol^ IP pulled down Sam68 specific protein band of 68 kDa and three additional Sam68 reactive bands. In addition to the proteins of expected size in the IP experiments, higher molecular weight Sam68 reactive bands in FMDV 3D^pol^ IP could be attributed to a shift due post-translational modifications of Sam68 [69]. The lower molecular weight bands of ~55 and ~25 kDa have been also reported by Lawrence et al., and discussed therein [13]. Alternatively, the known adaptor function of Sam68 that bridges two or more proteins of importance in cell signaling [72, 74] may contribute to the binding of 3D^pol^ and other cellular proteins to Sam68 thus producing the higher molecular weight protein bands. The right panel of Fig. 6a corresponds to results of an anti-Sam68 IP and the corresponding controls examined by Western blot for the presence of FMDV 3D^pol^. The isotype antibody IP (lane 1) and mock-infected cell lysates (lane 2) showed no 3D^pol^ specific band. In contrast, lane 3 (FMDV-infected cell lysates control) and lane 4 (anti-Sam68 IP) showed 3D^pol^ specific bands. Together, the reciprocal pulldown experiments of Sam68 and FMDV 3D^pol^ from FMDV-infected cell lysates provides the evidence for Sam68 and FMDV 3D^pol^ interaction in virus-infected cells.

In an effort to determine the region of 3D^pol^ that interacts with Sam68, we probed for binding of Sam68 in an ELISA using a 3D peptide fragment library consisting of fragments of about 50 amino acids (aa) in length starting from the N-terminus of 3D^pol^ (frag-1: aa 1–48, frag-2: aa 49–108, frag-3: aa 109–157, frag-4: aa 158–217, frag-5: aa 218–268, frag-6: aa 269–331, frag-7: aa 332–404, frag-8: aa 405–470). As evident from the data, frag-4 and frag-8 of 3D^pol^ bind to Sam68 with high affinity whereas frag 2, 6 and 7 bind to Sam68 with low affinity (Fig. 6b). This experiment provides evidence that the binding of Sam68 observed in the FMDV infection pulldown is due to binding to 3D^pol^ involving amino acid residues found in frag-4 and frag-8.

Furthermore, we used molecular docking software to illustrate the putative interaction between 3D^pol^ and Sam68 (Fig. 6c). Panels i and ii show the electrostatic surfaces of 3D^pol^ and Sam68, respectively, in binding pose. The electronegative surface reflected in the form of red colored bottom surface of 3D^pol^ and blue electropositive surface of Sam68 appear to participate a strong electrostatic attraction between the two molecules shown in panel iii. Panel iv shows the docked 3D^pol^ (green)-Sam68 (blue) complex in cartoon mode. Frag-4 residues 193–217 shown in orange color and frag-8 residues 453–470 in red color form the major Sam68 binding interface in 3D^pol^. Although, frag-5 residues 221, 222, 225, 226 also align the Sam68 binding interface, the major participation in the binding comes from frag-4 and 8, consistent with the peptide library ELISA data, further reinforcing the importance of these two regions in FMDV 3D^pol^ in interaction with Sam68. The fact that Sam68 has been shown to interact with other picornaviruses and that 3D^pol^ structure is largely preserved among picornaviruses, there might exist a common mechanism by which Sam68 affects picornavirus replication. Together the data from IP, ELISA and computational docking suggest that Sam68 binds to FMDV 3D^pol^ including residues contained in frag-4 and frag-8.

Finally, given our previous report indicating the cleavage of Sam68 by FMDV 3C^pro^ as infection progressed, we wanted to ascertain if Sam68 could be similarly co-precipitated with 3C^pro^ as was observed with 3D^pol^. Mock-infected or FMDV-infected cells at a MOI of 10 were lysed at 1, 3, and 5 hpi. The resulting cell lysates were subject to IP with mouse monoclonal anti-FMDV 3C^pro^, and the eluates thereof were probed with rabbit polyclonal anti-Sam68 (N-terminal) on a Western blot. A strongly reacting band at 68 kDa was detected at 5 hpi with a few lower molecular weight fragments faintly reacting, as well as a faint 68 kDa band at 3 hpi (Fig. 6d, left panel). To determine if the co-precipitation of Sam68 and 3C^pro^ was coincident with the appearance of cytoplasmic Sam68 degradation products, the cytoplasmic fraction of mock-infected and FMDV-infected cells at 1, 3, and 5 hpi were examined by Western blot probing with anti-Sam68 (N-terminal), which revealed the appearance of Sam68 cleavage fragments at 5 hpi coincident with the binding interaction between Sam68 and 3C^pro^ (Fig. 6d, right panel). It is noteworthy that under the conditions of this assay we cannot rule out the possibility that Sam68 may bind the unprocessed 3CD precursor protein through it predicted interaction with 3D^pol^. Also, these findings are in agreement with those presented in Fig. 1, where partial re-localization of Sam68 from the nucleus to the cytoplasm is detected in cells also staining positive for FMDV proteins. We conclude that the nucleocytoplasmic movement of Sam68 and its subsequent interaction with 3C^pro^ and 3D^pol^ are important steps in the progression of FMDV infection.

## Discussion

Picornaviruses are small, cytoplasmic, positive-sense RNA viruses with a simplified life cycle. The organization of cellular organelles and the sub-cellular localization of proteins change as picornavirus infection progresses. Such changes influence virus replication as well as cellular processes such as, trafficking along the membranes of these compartments and evasion to the host immune responses [75, 76]. Among known host proteins that appear to be involved in the progression of virus infection, Sam68 exhibits increased cytoplasmic accumulation in host cells infected with picornaviruses including: PV, FMDV, EV-71, and HRV [13, 14, 76–78]. In this study, we examined the nucleocytoplasmic movement of Sam68 and its interaction with viral factors to gain a better understanding of its role in the progression of FMDV infection.

Viruses shutoff the host protein translation and exploit the cell translational machinery for the maximal translation of their own (viral) proteins and to evade the host defense mechanisms [79]. The assembly of ribosomal initiation complex and ancillary factors on the IRES precedes the ribosomal scanning for initiation codon in FMDV RNA. Many cellular proteins reportedly bind the FMDV IRES but only a handful has been demonstrated to play a role in translation [80–82], suggesting that this is still an unresolved observation. The evidence presented in the current study suggests that RNA containing RAAA sequences in FMDV IRES domains 3 and 4 are important for binding of Sam68. In EMSA competition experiments, both IRES domain 3 and 4 of IRES (added in 10-fold molar excess) were capable of fully disrupting the RNP complexes formed by labeled domain 4 probe and Sam68. It could be argued that the interference caused by IRES domain 3 (with CAAA motif in its apical region) could be the result of a direct competition for Sam68 binding or be due to distant RNA-RNA interactions causing a disruption on complexes formed between Sam68 and IRES domain 4. We cannot completely exclude the possibility of Sam68 involvement in stabilizing or modulating long-range IRES RNA-RNA interactions between domain 3 and 4 [12, 83, 84]. Consistent with our findings, Lin et al. [36] had reported that UAAA and to less extend the RAAA sequences are suitable binding motifs for Sam68 protein. It is important to note that the nucleic acid binding cleft of the KH domain containing RNA binding proteins can accommodate only four unpaired bases [85]. Indeed, the RAAA sequence motifs in FMDV IRES domains 3 and 4 described herein are predicted to be contained in stretches of very small-unpaired RNA segments and could perfectly be accommodated in the RNA binding cleft of Sam68 KH domain. This is also supported by earlier evidence showing that Sam68 preferentially binds to single-stranded UAAA RNA motifs as evidenced by SELEX analysis and also within the 3′ NTRs of retrovirus transcripts as well as certain cellular mRNAs [13, 36, 86, 87].

In light of the above-mentioned observations, we sought to explore the functional significance of the Sam68-FMDV IRES interaction in more detail. To that end, we first utilized cell-free *in vitro* translation-RNA replication assays, which have proven to be instrumental in defining the role of many individual host or virus proteins at the molecular level [88–91]. Our *in vitro* cell-free experiment using Sam68-depleted extracts, revealed a defect in FMDV RNA synthesis, but not a significant reduction on virus translation, which we did not anticipate based on earlier studies [13, 92]. We believe that other splice variants of Sam68 including SLM-1 and SLM-2 (see Results section and Additional file 1: Figure S1), which contain an intact KH domain, but lack the Sam68-N-terminus, are still present in the depleted extracts, and could potentially bind to the FMDV IRES. It is possible that programming of cell fee translation reactions with high RNA concentrations (500 ng per reaction) could have led to similar end-point detection of 3D^pol^ by Western blot. Future studies using lower RNA concentration and shorter incubation times to program FMDV protein synthesis in CFEs could help resolve this issue.

Results obtained in our characterization of mutated forms of genetically engineered G-luc replicons, as well as viral genomes, provide compelling evidence for the significance of the RAAA motifs in domain 3 and 4 in FMDV IRES-driven translation. It is noteworthy that the full genome and replicon mutants exhibited impairment in their translation and replication. Interestingly, our earlier studies showing reduction in FMDV titers by Sam68 siRNA knockdown and the results described herein, suggest that it is possible that subtle modifications in the Sam68 interaction with the FMDV IRES could impact other functions that this protein exerts that are needed for efficient virus replication. This supposition is in line with multi-functional properties attributed to Sam68 (see Background). Indeed, Sam68 exhibits specific binding to FMDV 3C^pro^ and 3D^pol^ in infected cells. PV 3D^pol^ has also been shown to interact with Sam68 [14]. Using an indirect ELISA assay and 3D^pol^ fragments, it was suggested that 3D^pol^ frag-4 (aa 158–217) and frag-8 (aa 405–470) bind Sam68 with high affinity. However, under the experimental conditions, we cannot exclude the possibility that frag-2 (aa 49–108), frag-6 (aa 269–331), and frag-7 (aa 332–404) could also provide a Sam68 binding interface. In fact, the docking poses of the electrostatic surfaces of FMDV 3D^pol^ [Fig. 6c (i)] and Sam68 [Fig. 6c (ii)] clearly indicate that the two proteins share a large interfacial area that could be shared by more than one domain in either protein. In particular, the Sam68 binding interface of 3D^pol^ is formed by aa 193–217 and aa 453–470 in frag-4 and 8 that are part of the functionally critical palm and thumb domains of 3D^pol^ Fig. 6c (i). The 3D^pol^ structure consisting of thumb, palm, finger and fingertips domains is conserved among picornaviruses. Another striking feature of the Sam68-3D^pol^ interaction is the charge complementarities between the binding surfaces of the two proteins (electro-negative of 3D^pol^ and electro-positive of Sam68). Further studies will be required to determine the significance of these protein interactions for viral infection.

The observation that Sam68 co-precipitates with both the FMDV 3C^pro^ and 3D^pol^ also raises additional questions regarding the FMDV-induced cleavage of Sam68. The FMDV 3D^pol^ and transiently expressed 3CD precursor are known to partially localize to the cell nucleus due to a nuclear localization signal in the N-terminus of 3D^pol^ [93–95]. This was the basis of our speculation that the coincident nuclear efflux of Sam68 with the observed FMDV-induced cleavage was due to the maturation of 3C^pro^ from nuclear-localized 3CD precursor [13]. The 3C^pro^ cleavage of host cell transcription factors found in the nucleus of PV-infected cells also supports this notion [54]. Therefore, given Sam68 can interact with both 3C^pro^ and 3D^pol^, it remains to be determined whether WT full-length Sam68 is cleaved by FMDV 3CD or fully matured 3C^pro^. Moreover, since we also observe by Western blot some accumulation of full-length Sam68 in the cytoplasm as FMDV infection progresses, it is undetermined whether the full-length or cleaved form of Sam68 contributes to the modulation in virus replication. Like the full-length Sam68, the 3C^pro^ cleaved Sam68 is predicted to maintain its RNA-binding KH domain. Potentially, the C-terminal cleavage eliminates steric hindrances allowing for tighter binding.

The significance of the appearance of Sam68 truncation products as FMDV infection progresses still remains to be determined, yet the significance of Sam68 to HIV infection has been highlighted by how a Sam68 isoform with a C-terminal deletion (Sam68DeltaC) potently inhibits the progression of HIV infection [17, 30]. Sam68DeltaC reportedly represses translation of HIV genes by several mechanisms including sequestration of HIV transcripts to perinuclear bundles (PBs) and SGs as well as blocking the association of PABP1 with the 3′ end of the viral transcripts [22, 23, 32, 61]. Thus, there are two interesting parallels between our findings and effects of Sam68 on HIV: first, that FMDV stimulates the association of Sam68 with at least one marker of SGs in the cytoplasm; and second, that Sam68 affects the synthesis of viral RNA *in vitro* in the cell-free system. Interestingly, Piotrowska et al. [16] postulated that Sam68 is a virus infection specific marker of SGs. Consistent with our findings, White et al. reported the formation and subsequent disappearance of SGs as a result of PV infection [96]. Given the translation and replication arrest attributed to RNA transcripts sequestered in SGs and that Sam68 potently stimulated FMDV RNA replication, it seems likely that the FMDV RNA is similarly excluded from SGs.

## Conclusions

Sam68 is a host factor co-opted by FMDV during infection of susceptible host cells. As infection progresses, Sam68 becomes partially redistributed to the cytoplasm, where earlier in infection, it co-localizes with markers of SGs. Interaction of Sam68 with the FMDV IRES appears to promote viral RNA translation *in vitro*. Disruption of the IRES RAAA motif leads to non-viable virus progeny and defective replicons, though this may not be attributable to Sam68 interaction with the IRES alone. Finally, consistent with a role in RNA replication and its observed cleavage during FMDV infection, Sam68 co-precipitates with both FMDV 3C^pro^ and 3D^pol^.

## Methods

### Materials

Stratagene QuickChange IIXL mutagenesis kit was purchased from Agilent Technologies, Inc. (Santa Clara, CA). Lipofectamine 2000 DNA transfection reagent and streptavidin-coupled magnetic beads were purchased from Invitrogen (Carlsbad, CA). Sam68 expression plasmids pGEX-2 T Sam68 (Containing GST-tagged Sam68), pcDNA3 HA-tagged Sam68-WT and pcDNA3 HA-tagged Sam68 delta-KH (Sam68-KH-del) were purchased from Addgene Cambridge, MA, USA. LightShift® Chemiluminescent RNA EMSA Kit and the NE-PER cellular fractionation kit were purchased from Thermo Fisher Scientific Inc. (Rockford, IL). Restriction enzymes EcoRI and BamHI were purchased from New England Biolabs (Ipswich, MA).

### Antibodies

Polyclonal rabbit anti-Sam68 directed against the C-terminus of Sam68 (C-20), polyclonal goat anti-TIA-1, and HA-probe Antibody (F-7) were purchased from Santa Cruz Biotechnology (Santa Cruz, CA). Mouse monoclonal anti-G3BP was purchased from Abcam (Cambridge, MA). Polyclonal rabbit anti-Sam68 directed against the N-terminus of Sam68 was purchased from Novus Biologicals (Littleton, CO). Monoclonal mouse anti-FMDV 3D^pol^ antibody was a gift from Dr. A. Clavijo at the National Centre for Foreign Animal Disease, Winnipeg, Manitoba, Canada. Rabbit polyclonal anti-VP1 sera were courteously provided by Dr. M. Grubman, ARS, USDA. Goat-anti-rabbit antibodies conjugated to AlexaFluor-488 (AF-488), donkey-anti-goat antibodies conjugated to AF-568 and goat-anti-mouse antibodies conjugated to AF-568 were purchased from Molecular Probes (Carlsbad, CA).

### Viruses, cell lines, plasmids and PCR primers

FMDV type A_24_ Cruzeiro/Brazil 1955 (A_24_ Cru, Gb, Acc #AY593768) was derived from the infectious cDNA clones pA_24_Cru [97]. FMDV A_24_ Cru carrying modified IRES (Table 1) with abrogated Sam68 binding sites were produced by site directed mutagenesis of full-length genome copy plasmid pA_24_Cru. The LFBK, LFBK-αvβ6 and IBRS2 cell lines were cultured in 10 % fetal bovine serum (FBS) in Dulbecco’s minimal essential media (DMEM) supplemented with antibiotic/antimycotic [55, 56]. Cells were grown at 37 ° C in a humidified 5 % CO_2_ atmosphere. All of the PCR primers used to generate different clones and mutations were purchased from Life Technologies (Carlsbad, CA). The primer sequences have been detailed in Table 1. GST-tagged Sam68 clone was purchased from Addgene (Cambridge, MA). Sam68-6H was prepared by sub-cloning the Sam68 coding gene between EcoRI and BamHI sites in pET30C+ vector from pGEX-2 T Sam68 for *E. coli* expression.

### Plasmid transfection

Mammalian cells were transfected with plasmid DNA using the Lipofectamine 2000 transfection reagent (Invitrogen) following the manufacturer’s protocol. Briefly, cells were grown to approximately 30–40 % confluence. The cell culture medium was replaced with serum-free media containing the desired plasmid and Lipofectamine 2000. Cells were incubated with the mixture for 48 h at 37 ° C. Post-incubation, the cells were harvested for examination by Western blot to confirm expression of the desired construct.

### Immunofluorescent microscopy (IFM)

Cells were grown on glass coverslips in 12-well plates until approximately 40 % confluence. Control uninfected cells were fixed immediately with 4 % paraformaldehyde [98] in PBS for 10 min (min) at room temperature (RT) prior to the introduction of virus into adjacent wells. After fixation of the control cells, the remaining wells containing cells on coverslips were infected with WT FMDV A_24_ Cru at a MOI of 10 and incubated at 37 °C for 1 h. Excess virus was removed by acid-washing the cells briefly followed by three rinses with virus growth media (VGM). Designated cells were then fixed with 4 % PF, representing a 1 hpi time point. Remaining cells were provided fresh VGM and incubated for 2, 3, 4, or 5 hpi at 37 °C, after which, they were fixed with 4 % PF. Cells were washed in PBS, permeabilized with 0.1 % Triton-X100 in PBS for 20 min on ice, and blocked with 3 % bovine serum albumin (BSA) for 30 min at RT. The cells were probed with primary antibodies at RT for 1 h. The following day the cells were incubated for 1 h at RT with goat-anti-rabbit and goat-anti-mouse conjugated with AF-488 or AF-568 (Molecular Probes), respectively. The cover slips were washed three times with PBS after each antibody treatment. After the final antibody treatment, the cover slips were air-dried and mounted onto glass slides with ProLong antifade medium supplemented with DAPI stain (Life Technologies). Cells were examined and images captured using a 100X oil immersion objective on an Olympus fluorescent microscope (Center Valley, PA). Images were refined and figures generated using Adobe Photoshop software (Adobe Systems Incorporated, San Jose, CA). When examining cells transfected with GFP-tagged Sam68, the permeabilization and subsequent antibody steps were omitted. Following fixation and PBS washes, the coverslips of the transfected cells were mounted using ProLong anti-fade with DAPI and examined as described above.

### Western blot

SDS-PAGE (sodium dodecyl sulfate-polyacrylamide gel electrophoresis) was carried out using a 12 % Nu-PAGE® pre-cast gel system (Invitrogen). Subsequently, the separated proteins were electro-blotted onto a nitrocellulose membrane (Sigma-Aldrich, Saint Louis, MO). After blocking the membrane with 5 % milk in PBS-T, specific proteins were detected with primary antibodies at indicated dilutions followed by goat-anti-rabbit or goat-anti-mouse antibodies conjugated with HRP (Sigma-Aldrich). Cellular α-tubulin, employed as an internal loading control protein, was detected with mouse monoclonal anti-tubulin antibody (1:10,000 dilution) that in turn was detected with (1: 20,000) goat anti mouse-HRP (Sigma-Aldrich). The bound HRP conjugate antibodies were reacted with the WestDura SuperSignal chemiluminescent reagent (Pierce) according to the manufacturer’s instructions and visualized on X-ray film (X-Omat; Kodak, NY).

### Cloning expression and purification of recombinant Sam68-6H

In order to generate hexahistidine (6H) fused-Sam68 (Sam68-6H), the Sam68 coding gene was sub-cloned into pET30c + plasmid from pGEX-2 T Sam68 plasmid using BamHI and EcoRI restriction sites. Rosetta-DE3 cells were transformed with Sam68-6H-pET30c + plasmid. Single colony containing the plasmid was inoculated in 100 mL LB broth containing 0.4 % glucose, 25 μg/mL kanamycin (K25) and 20 μg/mL chloramphenicol (C20), and grown at 30 ° C with 220 rpm rotor speed in a Thermo Scientific MaxQ 4000 benchtop orbital shaker. This overnight culture was used as a starter culture for 1 l (L) LB K-25, C-20 broth at 37 ° C. At an OD of 0.8, the culture was induced with 1 mM IPTG for 3 h. The cell pellets were harvested by centrifugation at 4000 rpm at 4 ° C in a Sorvall high-speed centrifuge. Sam68-6H was lysed for 20 min at RT with intermittent stirring in BugBuster™ cell lysis reagent supplemented with pepstatin and leupeptin. To disrupt the cellular nucleic acids cell lysate was then sonicated at 85 % slope for 5 min at 30 s intervals. After centrifugation of the lysates for 30 min at 12,500 rpm at 4 ° C, the supernatant was mixed with 5 mL Ni-NTA agarose slurry pre-equilibrated with 6H-binding buffer (Pierce) and incubated at RT with gentle shaking. This slurry was packed in a polypropylene column and eluted with 5 column volumes of 6H-elution buffers (Pierce). The fractions with highest protein purity were pooled and dialysed against 50 mM Tris–HCl pH 8.0 containing 10 mM β-mercaptoethanol, 10 % glycerol, pepstatin and leupeptin. Protein concentration was monitored against reagent blank at 280 nM and the identity of protein was confirmed by Western blot using 1:500 rabbit polyclonal antibody raised against C-terminus of Sam68 (Santa Cruz Biotechnology, C-20).

### Expression and purification of HA-tagged Sam WT and Sam68-KH-del

HEK-293 cells containing SV40 T-antigen (293 T cells) were either transfected with pcDNA3 HA-tagged Sam68-WT or pcDNA3 HA-tagged Sam68 delta-KH (Sam68-KH-del) using Lipofectamine-2000 reagent. After 48 h, the cells were lysed by incubating for 5 min in 1X NP40 lysis buffer containing 50 mM Tris–HCl, pH 8, 150 mM NaCl and 1 % NP40. Following lysis, the debris were removed by centrifuging the lysate at 15,000 rpm at 4 ° C and 20 min. HA-tagged Sam68-WT or Sam68-KH-del were purified using HA-tagged protein purification kit (MBL International, IL, USA) as per manufacturer’s instructions. The purified proteins were diluted to 10 times their initial volume in 1X RNA-EMSA buffer (Thermo Fisher Scientific, USA) and concentrated using Amicon Ultra-15 Centrifugal Filter Units with 30 KDa cutoff (EMD Millipore, USA). The identity of proteins was confirmed by Western blot probing with HA-probe antibody (F-7, Santa Cruz Biotechnology). Purified HA-tagged Sam68-WT and Sam68-KH-del were stored at −80 ° C until further use.

### Synthesis of FMDV 5′ NTR RNAs

FMDV A_24_ Cru infectious clone linearized with restriction enzyme SwaI was utilized as template for generating various IRES domain RNAs. Full-length IRES was amplified with primer pair P1579 S IRES -T7 and P1580 AS IRES. Various IRES fragments were amplified with their respective primer pairs as following: P1329 A_24_-T7cre and P1330 AS cre (cre-fragment); sense (S) 5′ NTR T7-P1287 and antisense (AS) 5′ NTR-P1288 (S-fragment); P21036 IRES loop 1-T7 and P21037 IRES loop 1 Reverse (IRES loop 1); P1584 IRES loop 2 Forward-T7 and P1585 IRES loop 2 Reverse (IRES loop 2); P21034 IRES loop 3-T7 and P21035 IRES loop 3 Reverse (IRES loop 3); P-1581-IRES-4 Forward-T7 and P-1582-IRES-4 Reverse (IRES loop 4). See Table 1 for the detailed description of primers. Phusion Hot Start II High-Fidelity PCR kit (Thermo Scientific) was used to amplify the IRES fragments. MEGAscript® T7 Transcription Kit (Life Technologies) was used to synthesize the IRES RNAs. Biotinylated IRES RNAs were generated using the same T7 transcription kit with the only difference being in terms of the nucleotide tri-phosphates (NTPs). For biotinylated IRES RNA, the unlabeled NTPs were replaced with equal concentration of Roche biotin-NTP mix. RNA fragments were purified using NucAway™ Spin Columns (Life Technologies). The RNAs were stored at −80 ° C until further use.

### Biotin RNA pull-down assay

Biotinylated FMDV IRES full length, domain 2, domains 2–4, and domain 4 RNA were transcribed using the Megascript T7 kit (Ambion) with rNTPs supplemented with biotin-11-CTP (Roche). First, 50 μL of streptavidin-M280 magnetic beads (Invitrogen) were pulled-down by a magnet and equilibrated in 1 mL of 1X binding and washing (B&W) buffer consisting of 5 mM Tris–HCl, pH 7.5, 0.5 mM EDTA, and 1 mM NaCl. Following another pulldown and removal of B&W buffer, 5 μg of recombinant Sam68 and 100 μg of tRNA were added to the beads and incubated at 37 ° C for 30 min in a final volume of 200 μL 1X B&W buffer. The beads with potentially bound RNA and protein were pulled-down and washed three times in B&W buffer. Finally, the beads were resuspended in 20 μL of 1X Laemmli sample buffer, heated at 98 ° C for 10 min, and the supernatant collected after centrifugation was loaded onto a 12 % SDS-PAGE gel followed by Western blot analysis probing with anti-Sam68 (Santa Cruz Biotechnology).

### Electrophoretic mobility shift assay (EMSA)

Sixty five nucleotide long 5′-biotin labeled synthetic RNA probe containing two potential Sam68 binding (UAAA) motifs in WT form (labeled as WT domain 4 probe) or mutated form (UACG) labeled as (mutant domain 4 probe) were purchased from Integrated DNA Technology (IDT, Coralville, IA) (See Table 1 for sequence description). The RNA binding reaction was carried out for both WT and mutant domain 4 probe for 20 min at RT in 30 μL reaction volume containing 50 mM Tris–HCl, pH 7.9, 100 mM NaCl, 10 mM MgCl_2_, 10 % glycerol, 1.0 mM DTT, 1.0 μg/μL tRNA and different concentrations of recombinant Sam68. Protein bound RNA was resolved from the free RNA on 6 % acrylamide gel in 0.5 X TBE buffers for 90 min at 60 volt (V) and 4 ° C, which was pre-run for 1 h at 60 V to maintain uniform current. RNA were transferred from gel to precharged Hybond nylon membrane at 30 V (constant voltage) for 45 min using NuPAGE® Novex®-trans-blot apparatus (Life Technologies) in the presence of 0.5 X TBE buffer. After the RNA were transferred to the nylon membrane, the RNA were crosslinked to the membrane using 2000 MJ-cm^−2^ ultraviolet radiation twice in Stratalinker UV-crosslinking system (Stratagene). The RNA was detected with LightShift Chemiluminescent EMSA Kit (Pierce) following the manufacturer’s instructions.

### Co-immunoprecipitation of FMDV 3C^pro^ and 3D^pol^ with Sam68

Following the manufacturer’s protocol, antibodies directed against FMDV 3C^pro^ or 3D^pol^ or Sam68 were separately bound and crosslinked to the Protein G Dynabeads (Novagen) using the reagents provided in the Pierce Crosslink Co-immunoprecipitation Kit (Pierce). BHK-21 cells uninfected or infected with FMDV for 4 h were lysed with 1X RIPA buffer (1 % NP40, 0.5 % sodium deoxycholate, 0.1 % SDS, and 1X PBS) supplemented with proteinase inhibitors and Benzonase™ (Novagen, Gibbstown, NJ). Approximately 1–5 mg of uninfected or virus-infected cell lysates were then mixed with different sets of antibody-coupled beads, washed, and eluted. IP eluates were mixed with Laemmli sample buffer [99] boiled, and analyzed by Western blot probing with antibodies against Sam68 (Abnova) or FMDV 3D^pol^.

### FMDV 3D^pol^ peptide library ELISA to determine Sam68 binding segment of 3D^pol^

A sandwich ELISA was performed where recombinant Sam68 was diluted in buffer carbonate-bicarbonate (pH 9.6) to obtain 0.05 μg/mL, and 100 μL/well was used to coat Nunc Maxisorp plates (Fisher Scientific). After overnight incubation at 4 ° C, the plates were washed on plate washer (SkanWasher 300 Version B) with 0.01 M PBS and 0.05 % Tween 20 (PBS-T). Next, individual 6His-tagged 3D^pol^ fragments were diluted to 10 ng/mL, whereas 6His-tagged full-length 3D^pol^ was diluted to 500 ng/mL in blocking buffer and added to the plate, followed by incubation at 37 ° C for 1 h on a rotary shaker followed by washing on plate washer (SkanWasher 300 Version B) with 0.01 M PBS and 0.05 % Tween 20 (PBS-T). One hundred μL of anti-6His was added at 1/500 dilution in blocking buffer followed by incubating the plate at 37 ° C for 1 h while shaking. The plate was washed using the plate washer (SkanWasher 300 Version B). One hundred μL of HRP (SureBlue-KPL) was added, and after about 10 min when the blue color developed in the positive wells, the reaction was stopped by adding 100 μL of SureBlue-KPL stop solution and read in the plate reader, Biotek ELx808 at 640 nm.

### Computational prediction and docking of 3D structure of FMDV 3D^pol^ and Sam68

For this purpose, first, the structural model of full-length Sam68 was made using Phyre-2.0 web server [100] and FMDV A_24_ 3D^pol^ using SWISS-MODEL [101]. These initial structures were energy minimized by hybrid molecular mechanics/molecular dynamics protocol in which first 1000 steps of energy minimization using replica exchange protocol. Finally, FMDV A_24_ 3D^pol^ and Sam68 were docked with Clus-Pro docking interface [102] and 20 binding poses were analyzed and the most common pose was selected for analysis of contact interface and bonding between the two proteins.

### Preparation of cell-free extract (CFE), Sam68 depletion, *in vitro* translation and synthesis of viral RNA

FMDV A_24_ RNA was transcribed from FMDV A_24_ plasmid linearized with the restriction enzyme SwaI. The CFE of BHK-21 cells were prepared with modifications to the protocol described earlier [88, 103]. Briefly, a 1.8 L culture of BHK-21 cells was harvested and lysed in the presence of 1.5 volumes of hypotonic K-HEPES buffer pH 7.4 to 1 volume of packed cells to the cell. The lysates were prepared, cleared of debris, and treated with micrococcal nuclease in the presence of 0.75 mM CaCl_2_ and 25 °C for 30 min. After this, the nuclease was inactivated with 3 mM EGTA, pH 8. Sam68 depletion was performed by mixing 100 μL of rabbit polyclonal anti Sam68 antibody (NBP1-80229, Novus Biological) with 1 mL of BHK-21 CFE and 50 μL Dynabeads protein G for 2 h at room temperature under gentle rotation. Following incubation, the mix was put on magnetic plate to separate the bead bound material (anti-Sam68 antibody-Sam68 protein). The clear supernatant was labeled as Sam68 depleted CFE, and used later for protein and RNA synthesis experiments. The FMDV A_24_ RNA synthesis, the effect of Sam68 on this process, and the detection of newly synthesized RNA were carried with following modifications to the protocol described by Pogany and Nagy [90]: 1) instead of using P^32^ labeled nucleotides, biotin-UTP was used to detect the newly synthesized RNA, 2) purified viral polymerase was used to initiate the polymerase reaction, and 3) the reaction was carried out at 37 ° C for 5 h. The newly synthesized RNA was spotted on pre-charged Hybond nylon membrane followed by detection of the signal with the Pierce nucleic detection kit following the manufacturer’s protocol. The translation reactions were carried out in CFE containing 20 amino-acyl tRNAs creatinine phosphate, creatine kinase, dTT, rATP, rGTP, rCTP, and rUTP at 32 ° C with aliquots collected at 30 min, 60 min, 90 min and 120 min, which were resolved on 4-12 % SDS gel, and detected by Western blot probing with anti-FMDV 3D^pol^.

### Construction of IRES mutant FMDV A_24_ Cru full genome and G-luciferase (G-luc) replicon plasmids

CAAA to CACG mutation in domain 3 was introduced by sub-cloning the regions with desired mutations I 3: CAAA to CACG, I 4: UAAA…..UAAA to UACG……UACG, and I 34: CAAA….UAAA……UAAA to CACG…UACG…..UACG mutations from synthetic DNAs containing the mutations spanning SanDI and XbaI sites (purchased from Biobasic Inc.). The parental A_24_Cru full genome plasmid clone was digested with restriction enzymes SanDI and XbaI and the fragments purified. Using Infusion primer pairs P-2182-Infusion-FMDV A_24_ IRES Forward and P-2183-Infusion-FMDV A_24_ IRES Reverse (Table 1), the mutant DNA was PCR-amplified with Phusion high fidelity PCR kit and subsequently SanDI/XbaI digested and cloned into the backbone using Infusion HD cloning kit (Clontech). The identities and integrity of the clones were confirmed by full genome sequencing.

### Construction of the A_24_ replicons containing the G-luciferase (G-luc) reporter

The IRES mutant FMDV replicons were created by substituting the B*stE*II-M*fe*I digested DNA fragments containing IRES mutations into a pA_24_ G-luc replicon system (Kloc et al., submitted as a separate manuscript). Briefly, these replicons were derived by replacing the L^pro^, and capsid coding sequences with a G-luciferase reporter gene, and later sub-cloning the IRES mutations into the A_24_ G-luc plasmid. The new constructs were labeled as: I3 G-luc, I4 G-luc and I34 G-luc. The RNAs were derived from either SwaI or MfeI digested plasmids and then transfected into BHK-21 cells using Lipofectamine 2000 transfection reagent (Life Technologies). The cell lysate obtained from samples collected at 0, 2, 6 and 24 hpt were clarified and the G-luc signal was assessed using a Microplate Luminometer. The G-luc signals were normalized to the amount total protein measured in each individual lysate. Two independent transfection experiments were conducted for each of the RNA and the G-luc values are expressed as relative luciferase units (RLU) per nanogram of protein.

## Additional file

Additional file 1: Figure S1. Sam68 truncated related proteins SLM-1 and SLM-2 maintain RNA-binding domains. **A.** Schematic of the alignment of full-length Sam68, Sam68-like molecule 1 (SLM-1), and SLM-2. The KH domain and the N- and C-terminal KH domains (NK, CK) embedded in the larger GSG domain is indicated. Also shown is the nuclear localization sequence (NLS), poly-tyrosine region (Poly Y), and multiple poly-proline regions (P1-P6). **B.** Clustal-Omega amino acid sequence alignment of human Sam68 (Acc #Q07666.1), SLM-1 (Acc #Q5VWX1.1), and SLM-2 (Acc #O75525.1). The RNA-binding KH domain is indicated by bolded and underlined letters and shaded background. (PPTX 56 kb)
