# Supplementary figures and images for: Analysis of the interaction between host factor Sam68 and viral elements during foot-and-mouth disease virus infections
Source: Virol J. 2015 Dec 23;12:224. doi: 10.1186/s12985-015-0452-8 (PMC4689063; doi:10.1186/s12985-015-0452-8)

## Slide 1
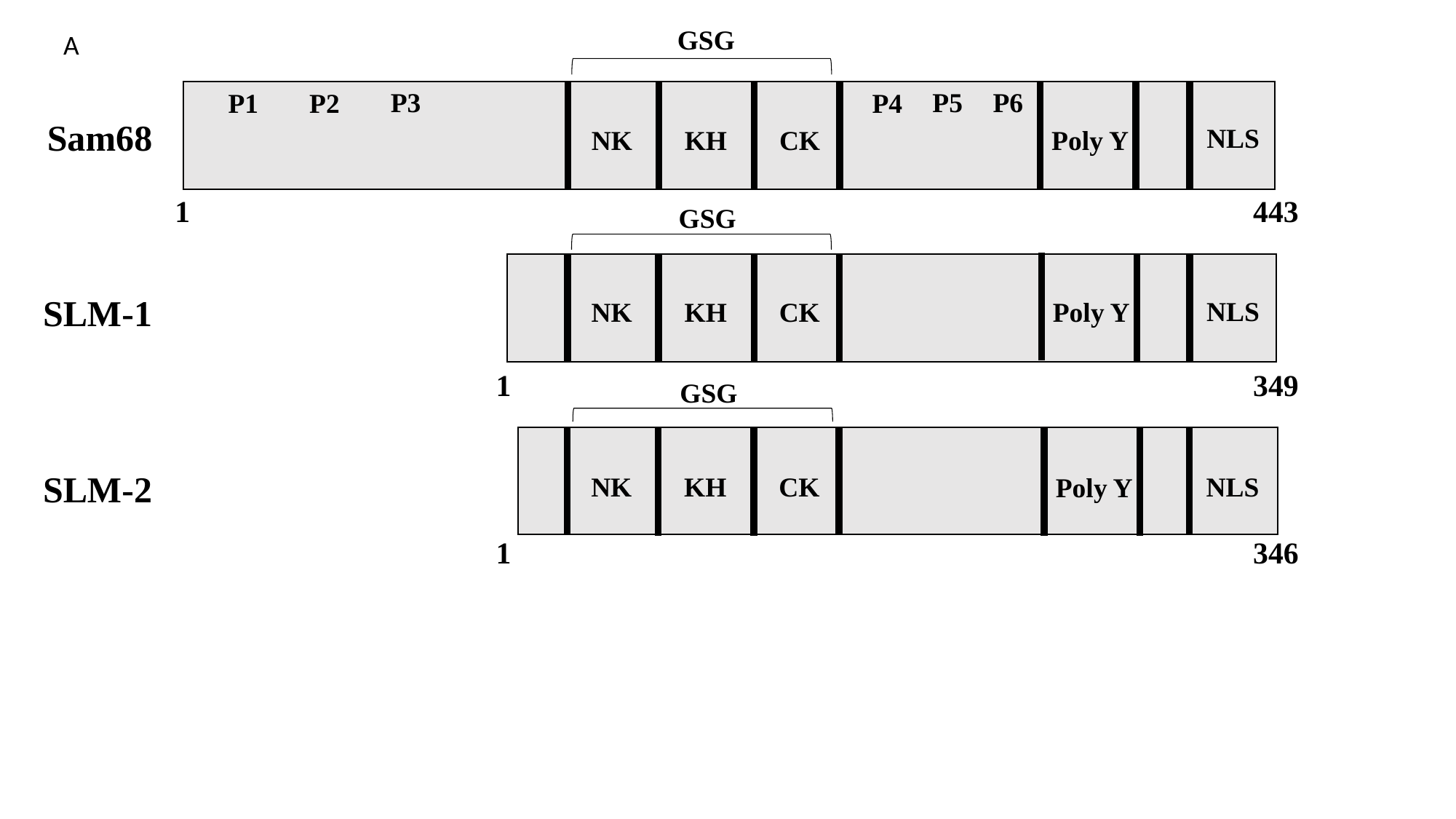

GSG
A
P6
P3
P5
P2
P4
P1
1
443
Sam68
NLS
NK
KH
CK
Poly Y
GSG
1
349
SLM-1
NLS
NK
KH
CK
Poly Y
GSG
1
346
SLM-2
NLS
NK
KH
CK
Poly Y

## Slide 2
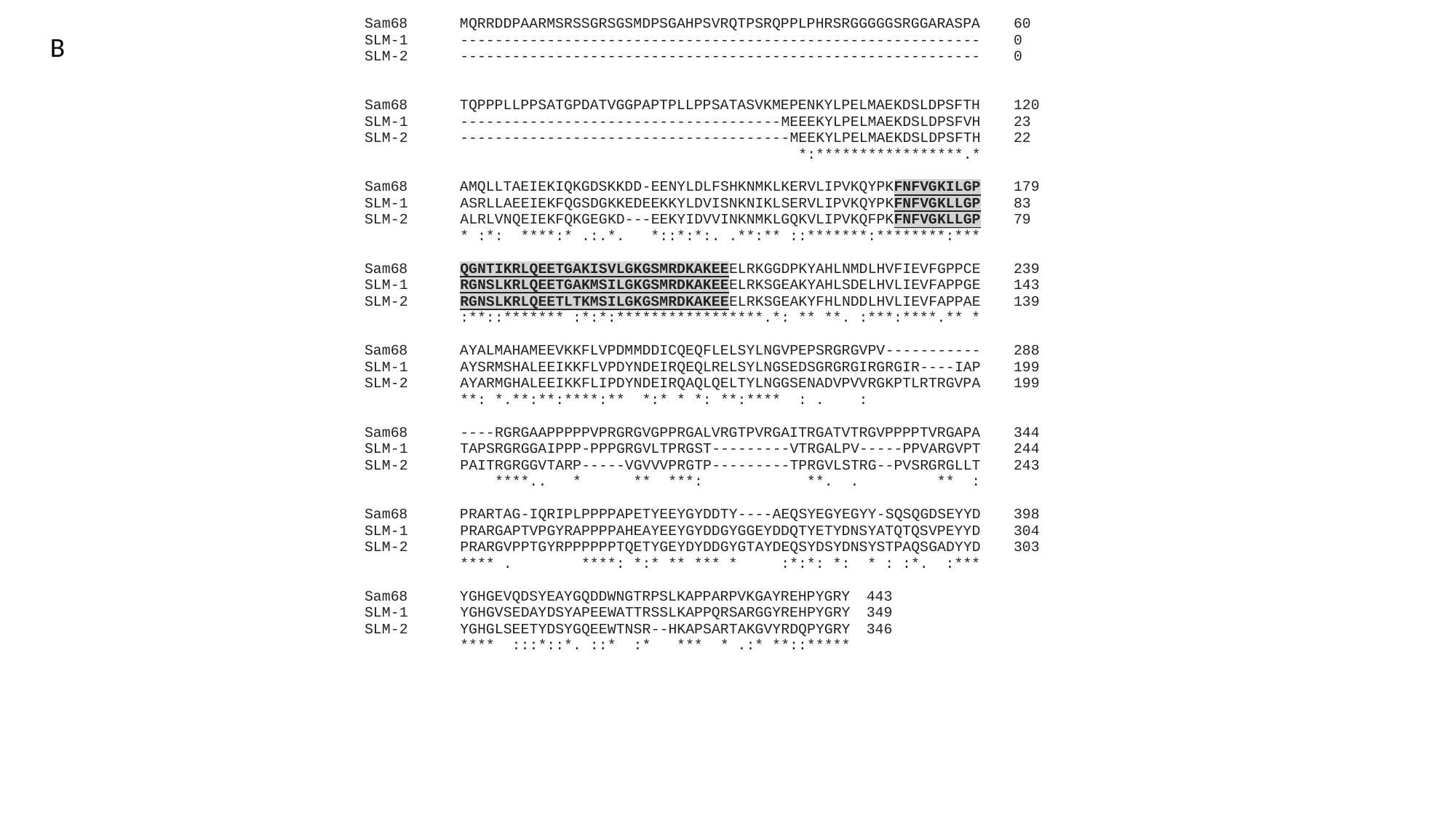

B

Supplement: Additional file 1: Figure S1. — Sam68 truncated related proteins SLM-1 and SLM-2 maintain RNA-binding domains. A. Schematic of the alignment of full-length Sam68, Sam68-like molecule 1 (SLM-1), and SLM-2. The KH domain and the N- and C-terminal KH domains (NK, CK) embedded in the larger GSG domain is indicated. Also shown is the nuclear localization sequence (NLS), poly-tyrosine region (Poly Y), and multiple poly-proline regions (P1-P6). B. Clustal-Omega amino acid sequence alignment of human Sam68 (Acc #Q07666.1), SLM-1 (Acc #Q5VWX1.1), and SLM-2 (Acc #O75525.1). The RNA-binding KH domain is indicated by bolded and underlined letters and shaded background. (PPTX 56 kb) [file 12985_2015_452_MOESM1_ESM.pptx]
